# P-2262. Outcomes of Patients with Severe Persistent BK Polyomavirus DNAemia

**DOI:** 10.1093/ofid/ofae631.2415

**Published:** 2025-01-29

**Authors:** Emily Eichenberger, Wairimu Magua, Geeta Karadkhele, Christian P Larsen

**Affiliations:** Emory School of Medicine, Atlanta, Georgia; Emory University, Atlanta, Georgia; Emory University School of Medicine, Atlanta, Georgia, Atlanta, Georgia; Emory University School of Medicine, Atlanta, Georgia

## Abstract

**Background:**

BK polyomavirus (BKPyV) is the leading cause of polyomavirus associated nephropathy (PyVAN) in kidney transplant recipients (KRT). The goal of this study was to determine risk factors and outcomes associated with severe persistent BKPyV (SPBK).

**Figure 1A:**
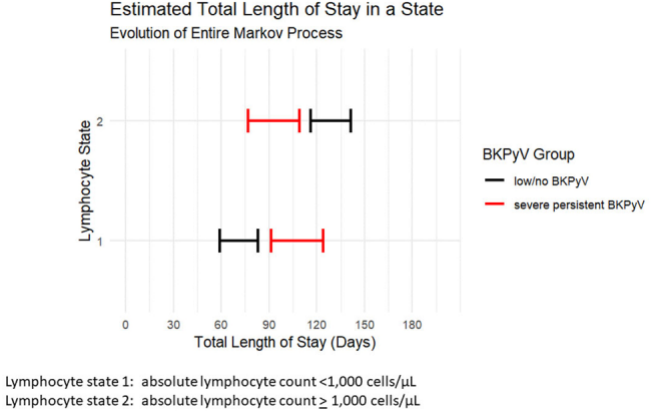
Total estimated length of stay in a lymphocyte state: SPBK vs LBK

**Methods:**

This is a single center, matched retrospective case control study of KTR with SPBK compared to 1) KTR with low/no BKPyV-DNAemia (LBK) and 2) KTR with high, transient BKPyV-DNAemia (HTBK). Definitions were as follows, SPBK: BKPyV load > 6 log10 for >90 consecutive days. LBK: BKPyV load remaining < 3 log10. HTBK: BKPyV load >4 log10 for < 90 consecutive days, all within the first 2 years post-transplant.

SPBK were exact matched 1:3 to LBK controls on race, sex, donor type immunosuppression protocol, and genetic matched with robust Mahalanobis distance on age. SPBK cases were exact matched 1:2 with HTBK controls on race, gender, immunosuppression protocol, and genetic matched with robust Mahalanobis distance on age and donor type.

Differences between case and control groups were analyzed using Wilcoxon-rank sum test and Fisher’s exact test. A multistate Markov model was used to evaluate whether time spent in a state of lymphopenia (< 1,000 cells/µL) was associated with SPBK.

**Figure 1A:**
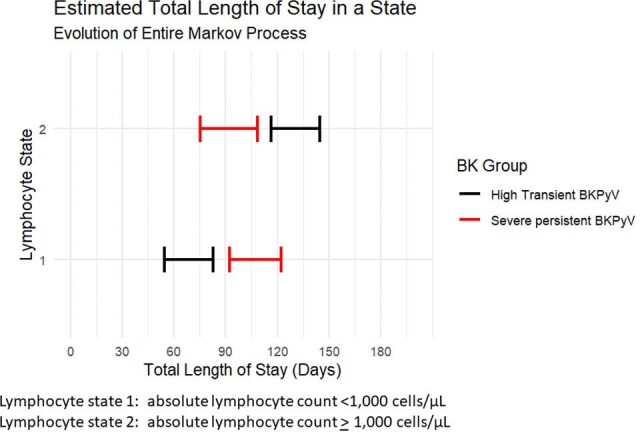
Total estimated length of stay in a lymphocyte state: SPBK vs HTBK

**Results:**

20 KTR with SPBK were matched to 60 LBK and 40 HTBK. Significantly more SPBK experienced acute cellular rejection within the first 2 years of transplant relative to LBK (75% vs 27%, p< 0.001) and HTBK (22%, p< 0.001). SPBK had a longer estimated duration of lymphopenia in the first 200 days post-transplant relative to LBK and HTBK (SPBK: 107.5 days, 95% CI 92.1, 122.7 vs LBK: 68.5 days, 95% CI 55.3, 84.8, vs HTBK 70.3 days 95% CI 58.8, 82.4, Figure 1A-B).

Median eGFR at 6 months post-transplant for SPBK was lower than patients with LBK and HTBK (44 vs 56, p=0.016; 44 vs 60, p=0.004, respectively). Burden of lab draws was significantly higher for SPBK vs LBK and HTBK (69 days vs 55, p< 0.001; 69 days vs 56, p=0.006, respectively). Total cost of labs was $7836 for SPBK vs $4,691 for LBK (p< 0.001) and $5922 for HTBK (p< 0.001).

**Conclusion:**

Patients with SPBK spent more time in a state of lymphopenia in the first 200 days post-transplant vs LBK and HTBK. SPBK is associated with worse renal function at 6 months post-transplant and is accompanied by a higher burden of lab draws and cost.

**Disclosures:**

Christian P. Larsen, MD, PhD, Bristol-Myers Squibb: Advisor/Consultant|CareDx: Advisor/Consultant|Eledon: Advisor/Consultant

